# Supplementary material for: Genomic analysis of T Cell receptors reveals lynch syndrome specific immune signatures
Source: Nat Commun. 2026 Apr 3;17:4790. doi: 10.1038/s41467-026-71243-z (PMC13219734; doi:10.1038/s41467-026-71243-z)
Supplement: Supplementary file 1 — Supplementary Information [file 41467_2026_71243_MOESM1_ESM.pdf]

Supplementary Information

Genomic Analysis of T Cell Receptors Reveals Lynch Syndrome Specific Immune Signatures

Nan Deng[1],#, Fahriye Duzagac[1],#, Ana M. Bolivar[1],#, Laura Reyes-Uribe[1], Melissa W. Taggart[2], Selvi Thirumurthi[3], Luigi Ricciardiello[3], Patrick M. Lynch[3], Y. Nancy You[4], Scott Kopetz[5], Paul Scheet[6], Gregory A. Lizee[7,8], Alexandre Reuben[9], Fatima Marin[10], Marta Pineda[10], Krishna M. Sinha[1], Ajay Bansal[11,12], Gabriel Capella[10], Eduardo Vilar[1,5]*

Contents

Supplementary figures 1-10

Supplementary tables 1-4

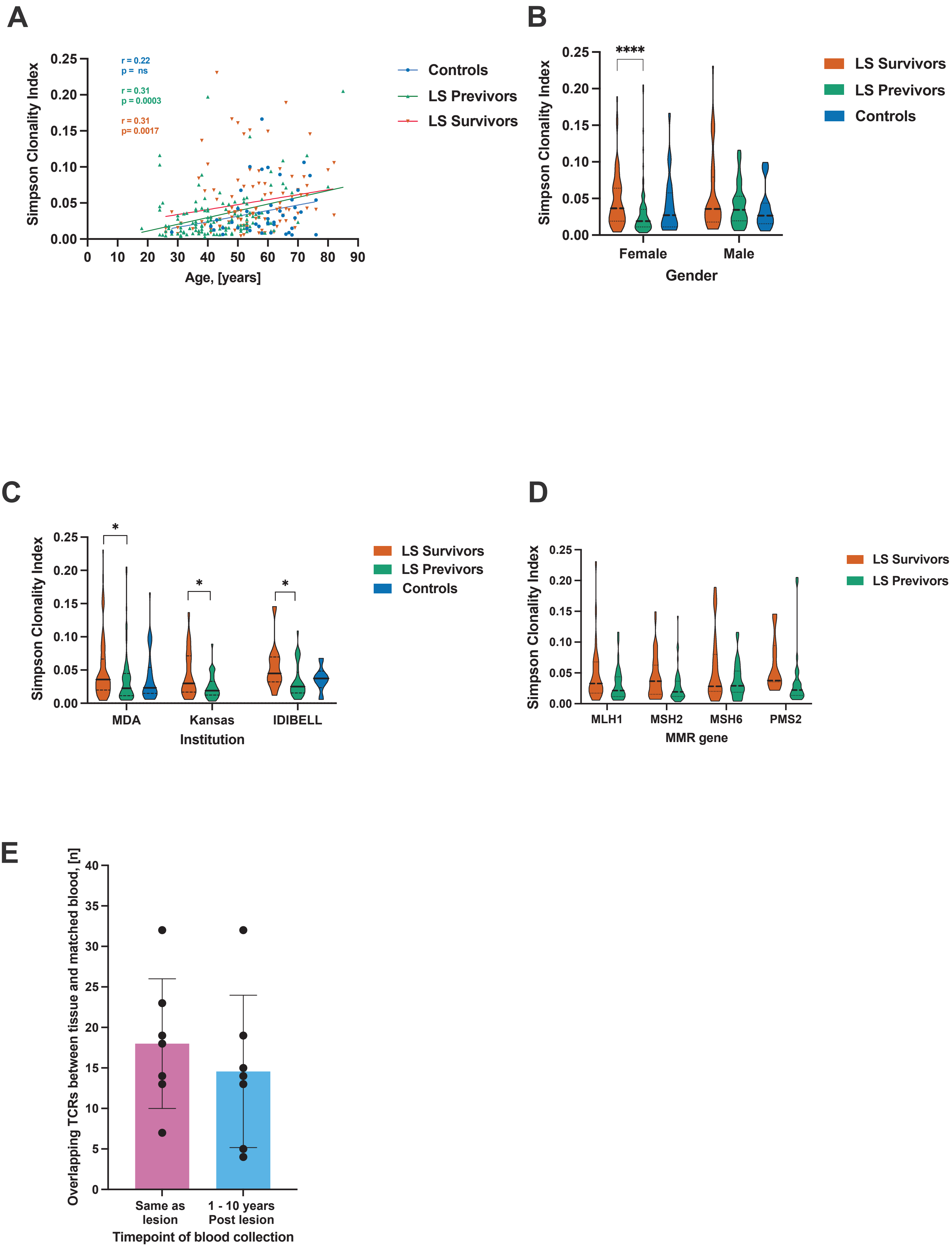
A
Simpson Clonality Index
Age, [years]
r = 0.22
p = ns
r = 0.31
p = 0.0003
r = 0.31
p= 0.0017
Controls
LS Previvors
LS Survivors
B
****
Simpson Clonality Index
Female
Male
Gender
LS Survivors
LS Previvors
Controls
C
Simpson Clonality Index
MDA
Kansas
IDIBELL
Institution
LS Survivors
LS Previvors
Controls
D
Simpson Clonality Index
MLH1
MSH2
MSH6
PMS2
MMR gene
LS Survivors
LS Previvors
E
Overlapping TCRs between tissue and matched blood, [n]
Same as lesion
1 - 10 years Post lesion
Timepoint of blood collection

Figure S1. Effect of demographic and clinical characteristics in the clonality of the TCR repertoires from peripheral blood. A, Age of the participants shows a positive weak correlation with the Simpson Clonality Index in survivors and previvors (Spearman *P*-value<0.01); **B,** Differences in the level of TCR clonality within the PBMCs/blood of female survivors previvors and controls as well as male survivors previvors and controls (Mann-Whitney *P*-value ****<0.0001); **C,** Differences in the level of clonality between survivors previvors and controls based on the institution from which the sample was collected (Mann-Whitney *P*-value *<0.05); **D,** Differences in the level of clonality between survivors and previvors based on the MMR gene in which they harbor the germline mutation – no statistical differences were observed; **E,** Differences in the number of overlapping TCRs between tissue samples and matched patient blood samples, based on whether the blood sample was collected at the same timepoint or at a different timepoint compared to the tissue sample collection. Source data are provided as Source Data file Source Data.xlsx.

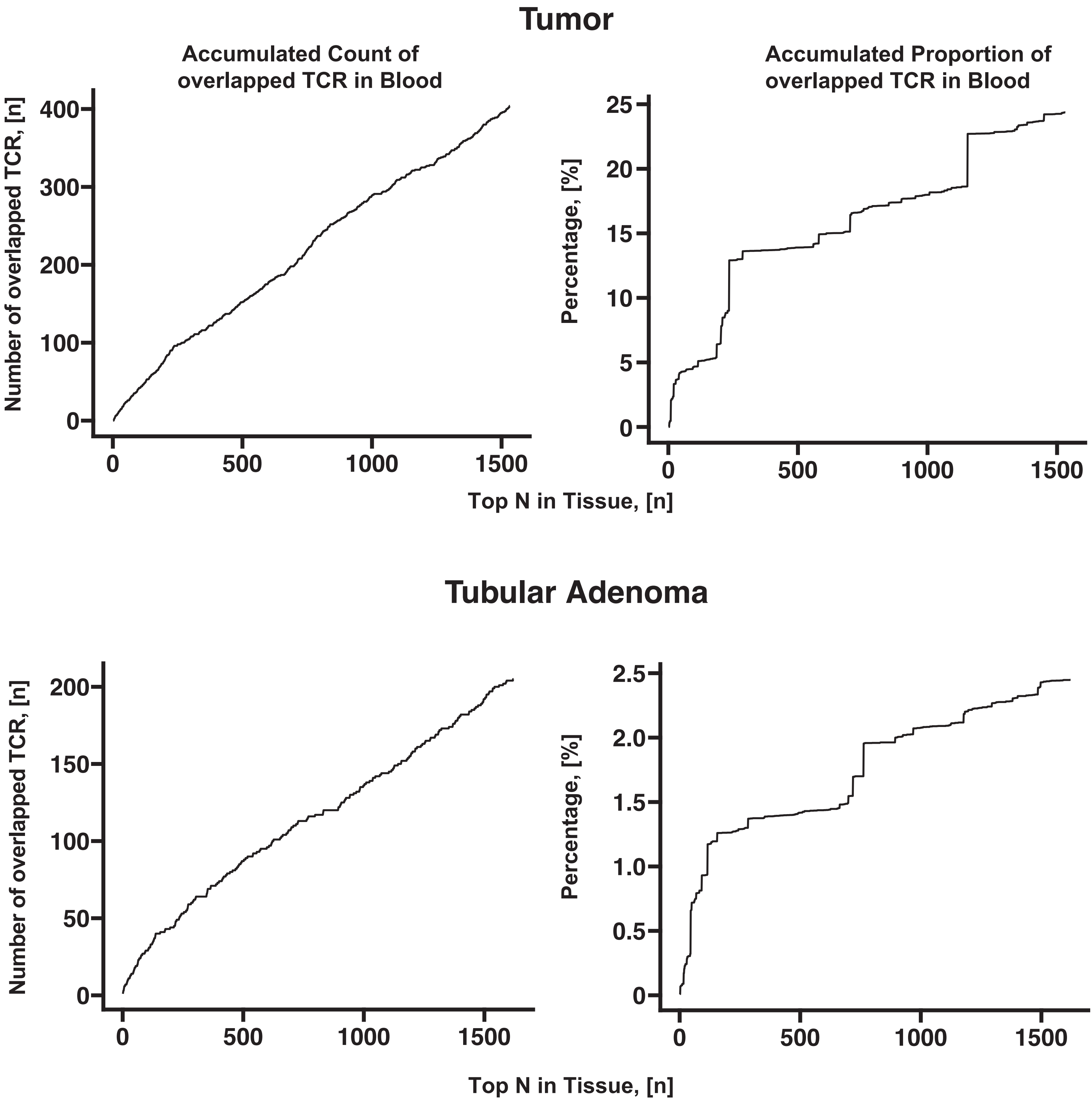
Tumor
Accumulated Count of overlapped TCR in Blood
Accumulated Proportion of overlapped TCR in Blood
Number of overlapped TCR, [n]
0
100
200
300
400
0
500
1000
1500
Percentage, [%]
0
5
10
15
20
25
Top N in Tissue, [n]
Tubular Adenoma
Number of overlapped TCR, [n]
0
50
100
150
200
Percentage, [%]
0
0.5
1.0
1.5
2.0
2.5
Top N in Tissue, [n]

Figure S2. TCRβ repertoire overlap between colorectal lesions and peripheral blood. A, Number of tumor-infiltrating TCRβs in colorectal cancer (CRC) that overlap with the top N expanded TCRβs in peripheral blood (Y-axis, left panel). **B**, Proportion of tumor-infiltrating TCRβs in tubular adenomas that overlap with the top N expanded TCRβs in peripheral blood (Y-axis, right panel). Source data are provided as Source Data file Source Data.xlsx.

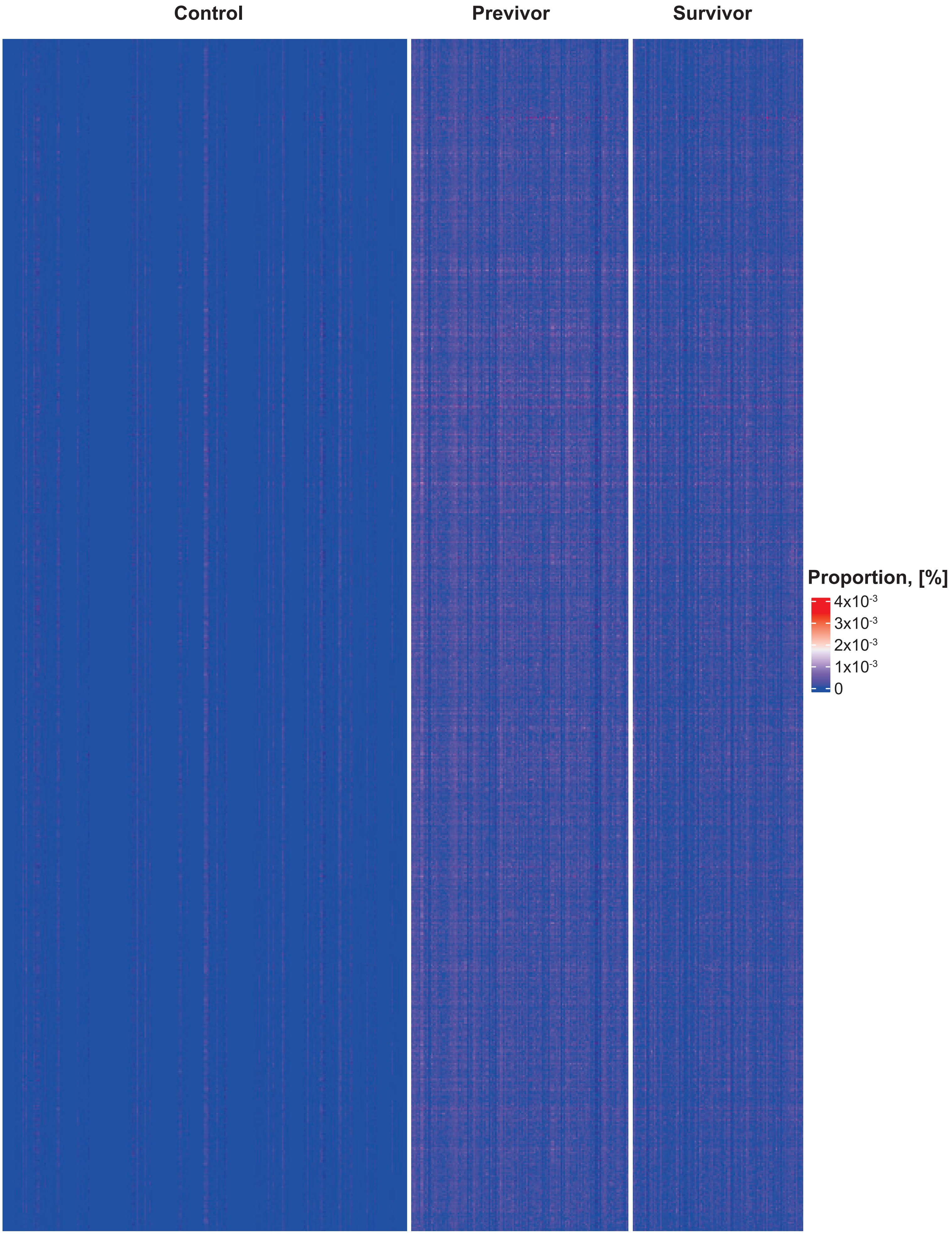

Control
Previvor
Survivor
Proportion, [%]
4x10^-3
3x10^-3
2x10^-3
1x10^-3
0

Figure S3. Heatmap showing the differences in frequency and expansion level of LS-associated TCRβs. Each column is a sample, and each row is a TCRβ. Yellow indicates higher expansion in the blood. Source data are provided as Source Data file Figure s3.xlsx.

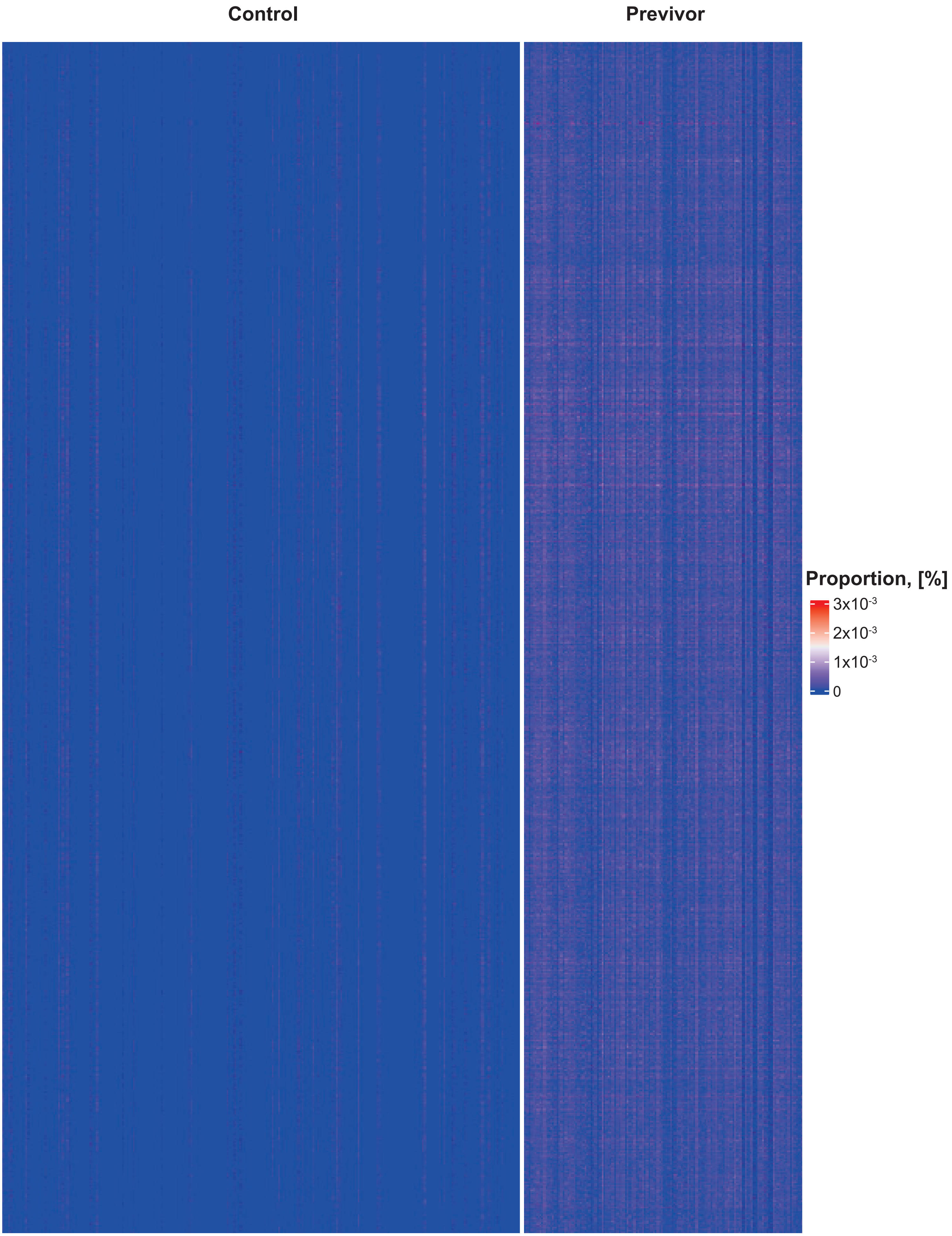
Control
Previvor
Proportion, [%]
3x10-3
2x10-3
1x10-3
0

Figure S4. Heatmap shows the differences in the frequency or level of expansion of the LS previvor-associated TCRβs. Each column is a sample, and each row is a TCRβ. Yellow indicates higher expansion in the blood. Source data are provided as Source Data file Figure s4.xlsx.

A

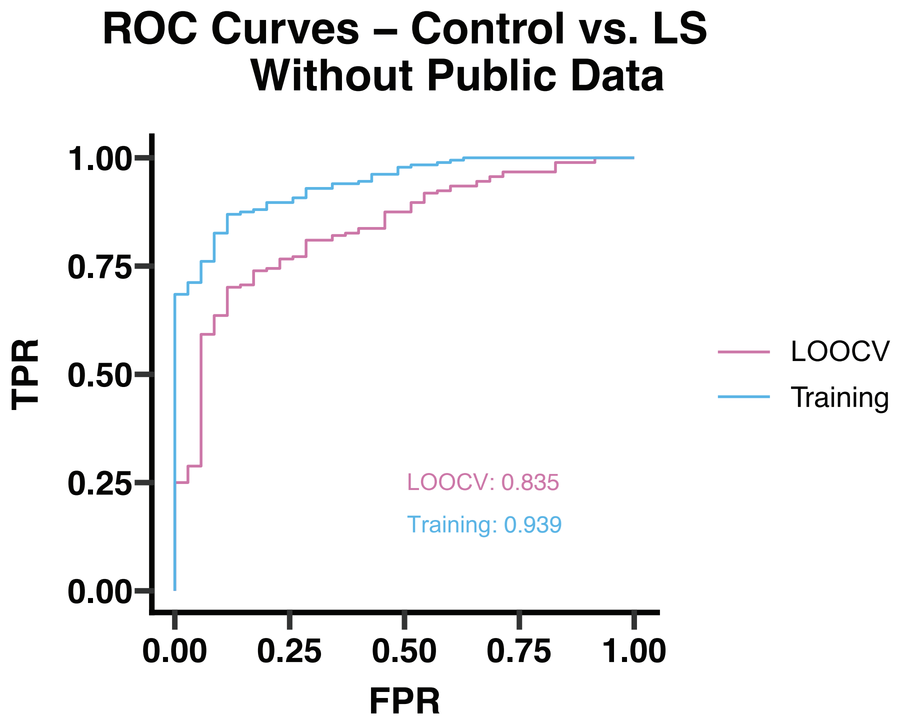

ROC Curves – Control vs. LS
Without Public Data
1.00
0.75
0.50
0.25
0.00
TPR
0.00
0.25
0.50
0.75
1.00
FPR
LOOCV: 0.835
Training: 0.939
LOOCV
Training

B

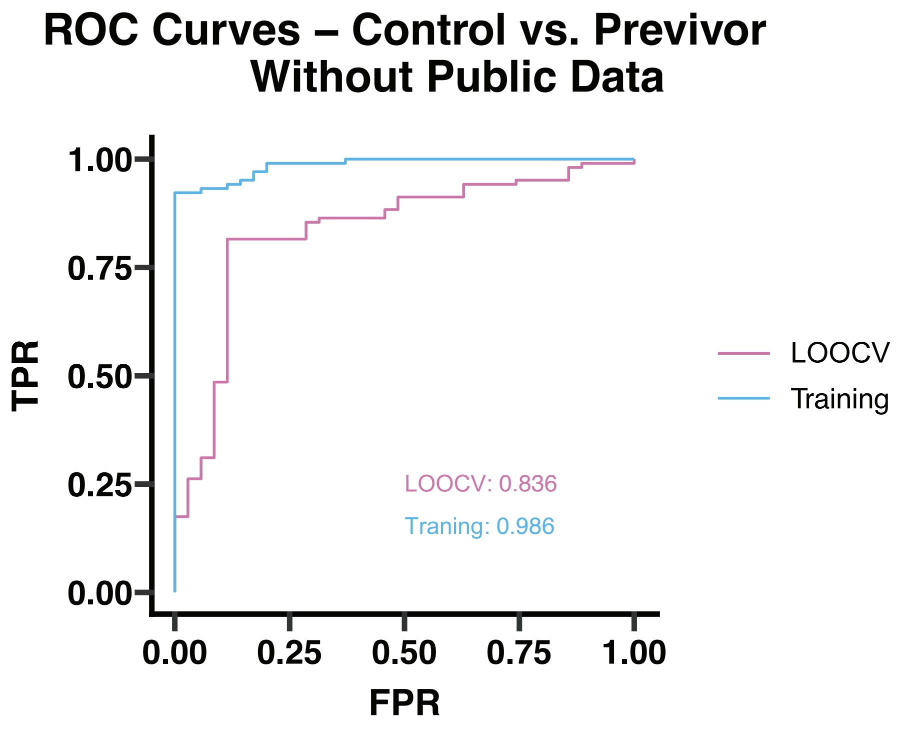

ROC Curves – Control vs. Previvor
Without Public Data
1.00
0.75
0.50
0.25
0.00
TPR
0.00
0.25
0.50
0.75
1.00
FPR
LOOCV: 0.836
Traning: 0.986
LOOCV
Training

Figure S5. Classification performance of LS-associated TCRβ signatures. Classification of PBMC/Blood samples as LS (Previvor + Survivor) vs. Control, and LS Previvor vs. Control, without balancing the negative controls using the public dataset; **A**. Area under the receiver operating characteristic curve (AUROC) showing the performance of LS-associated TCRβ signatures in classifying samples as LS or Control in the training and validation datasets. The best performance was achieved under the condition of positive ratio cutoff = 0.5, Fisher test cutoff = 10^{-4}, and Wilcoxon test cutoff = 10^{-3}; **B**. AUROC showing the performance of LS-associated TCRβ signatures in classifying samples as LS Previvor or Control in the training and validation datasets. The best performance was achieved under the condition of positive ratio cutoff = 0.1, Fisher test cutoff = 10^{-3}, and Wilcoxon test cutoff = 10^{-3}. TPR, true positive rate; FPR, false positive rate. Source data are provided as Source Data file Source Data.xlsx.

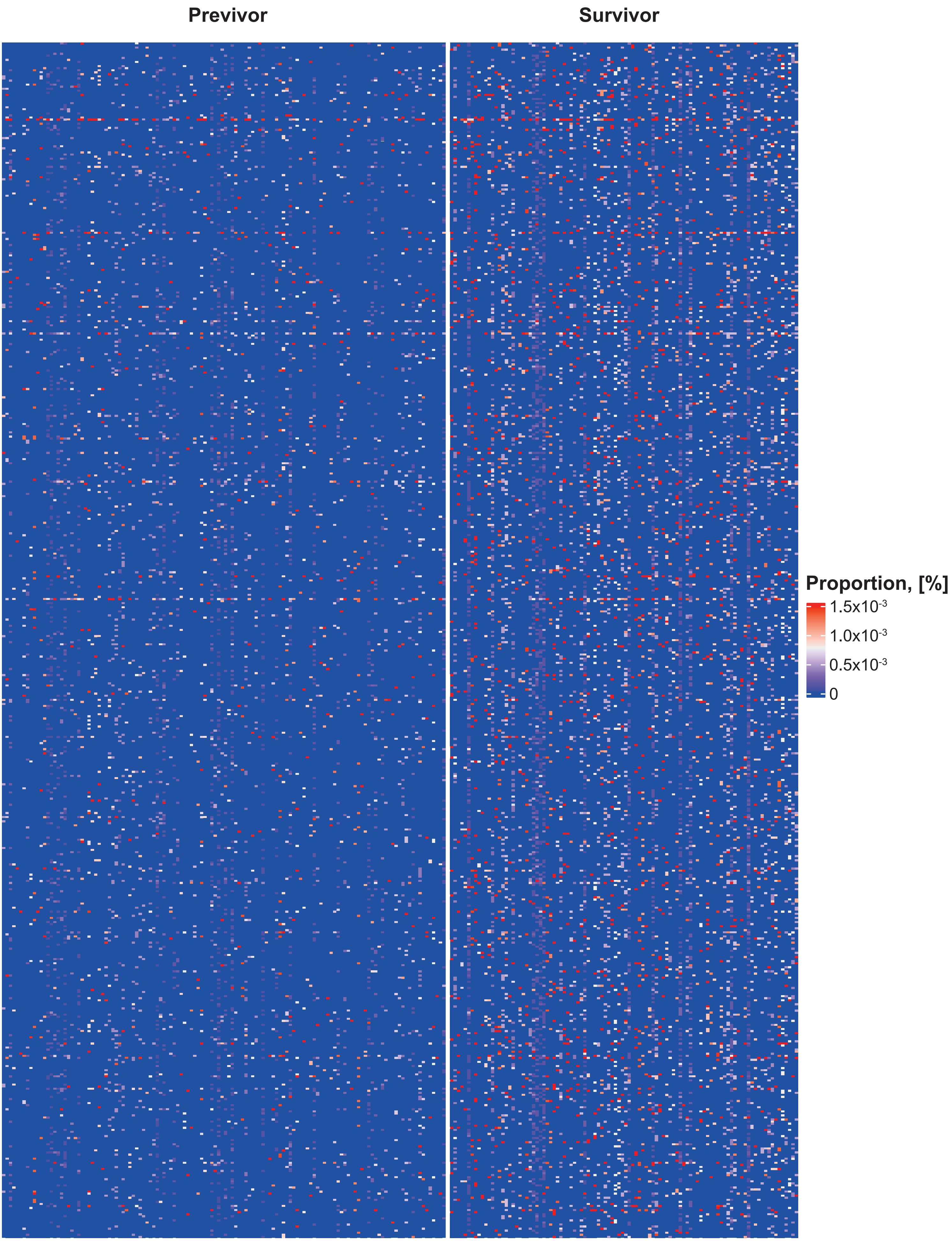

Previvor
Survivor
Proportion, [%]
1.5x10-3
1.0x10-3
0.5x10-3
0

Figure S6. Heatmap shows the differences in the frequency or level of expansion of the LS survivor associated TCRβs. Each column is a sample, and each row is a TCRβ. Yellow indicates higher expansion in the blood. Source data are provided as Source Data file Figure s6.xlsx.

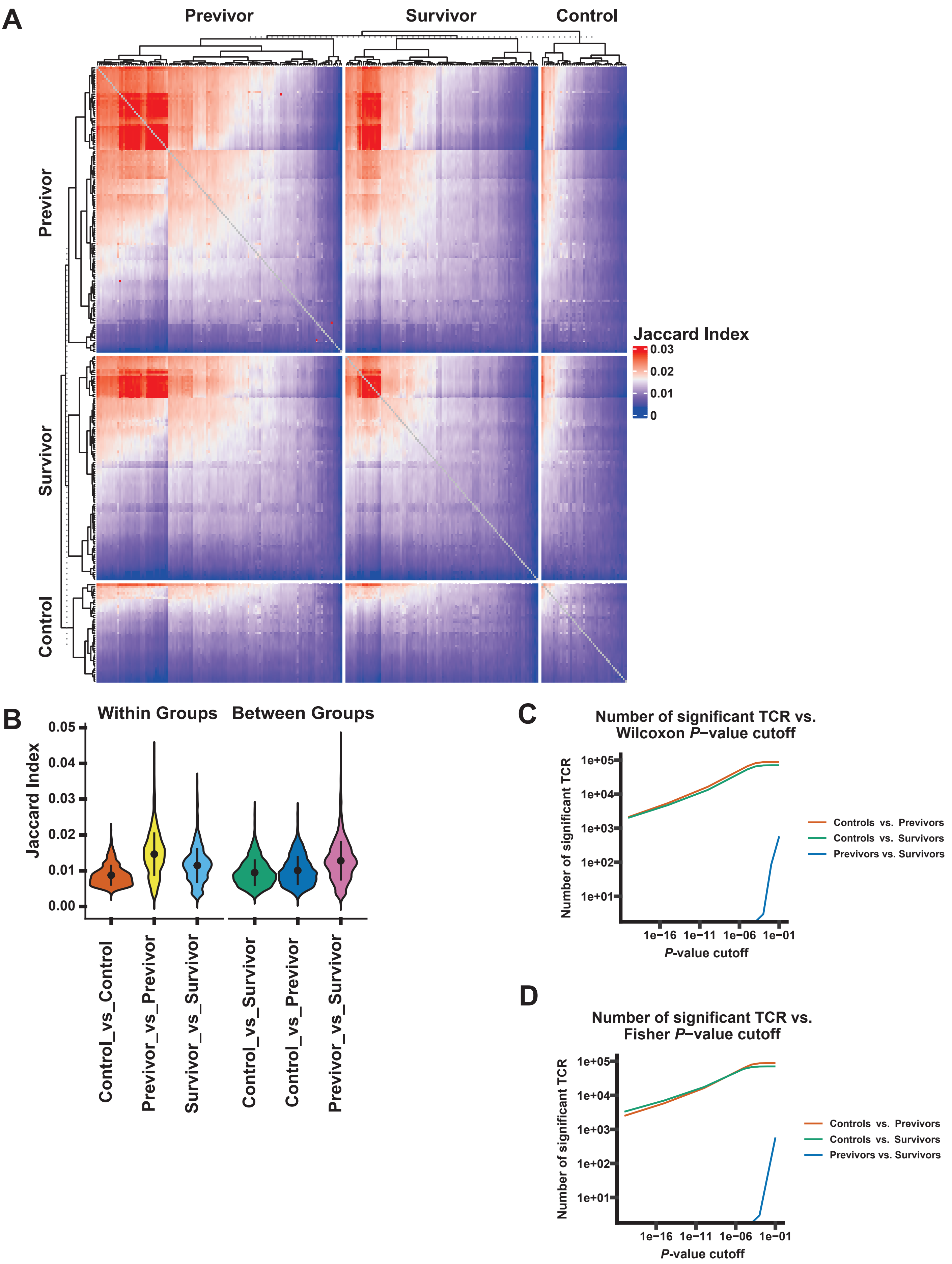
A
Previvor
Survivor
Control
Jaccard Index
0.03
0.02
0.01
0
B
Within Groups
Between Groups
Jaccard Index
0.05
0.04
0.03
0.02
0.01
0.00
Control_vs_Control
Previvor_vs_Previvor
Survivor_vs_Survivor
Control_vs_Survivor
Control_vs_Previvor
Previvor_vs_Survivor
C
Number of significant TCR vs.
Wilcoxon P−value cutoff
Number of significant TCR
1e+05
1e+04
1e+03
1e+02
1e+01
1e−16
1e−11
1e−06
1e−01
P-value cutoff
Controls vs. Previvors
Controls vs. Survivors
Previvors vs. Survivors
D
Number of significant TCR vs.
Fisher P−value cutoff
Number of significant TCR
1e+05
1e+04
1e+03
1e+02
1e+01
1e−16
1e−11
1e−06
1e−01
P-value cutoff
Controls vs. Previvors
Controls vs. Survivors
Previvors vs. Survivors

Figure S7. Similarity among healthy controls, LS previvors, and LS survivors. A. Heatmap of pairwise Jaccard distances based on TCRβ repertoires. Samples are grouped by cohort: healthy controls, LS previvors, and LS survivors; **B**, Violin plots showing the distribution of Jaccard distances for within-group comparisons (control–control, previvor–previvor, survivor–survivor) and between-group comparisons (control vs. previvor, control vs. survivor, and previvor vs. survivor); **C**, Spaghetti plot showing the number of significantly different TCRs (y-axis; abundance-based comparison using Wilcoxon test) across different significance thresholds (x-axis) for control vs. previvor (red), control vs. survivor (green), and previvor vs. survivor (blue); **D**, Spaghetti plot showing the number of significantly different TCRs (y-axis; incidence-based comparison using Fisher's exact test) across different significance thresholds (x-axis) for control vs. previvor (red), control vs. survivor (green), and previvor vs. survivor (blue). Source data are provided as Source Data file Source Data.xlsx.

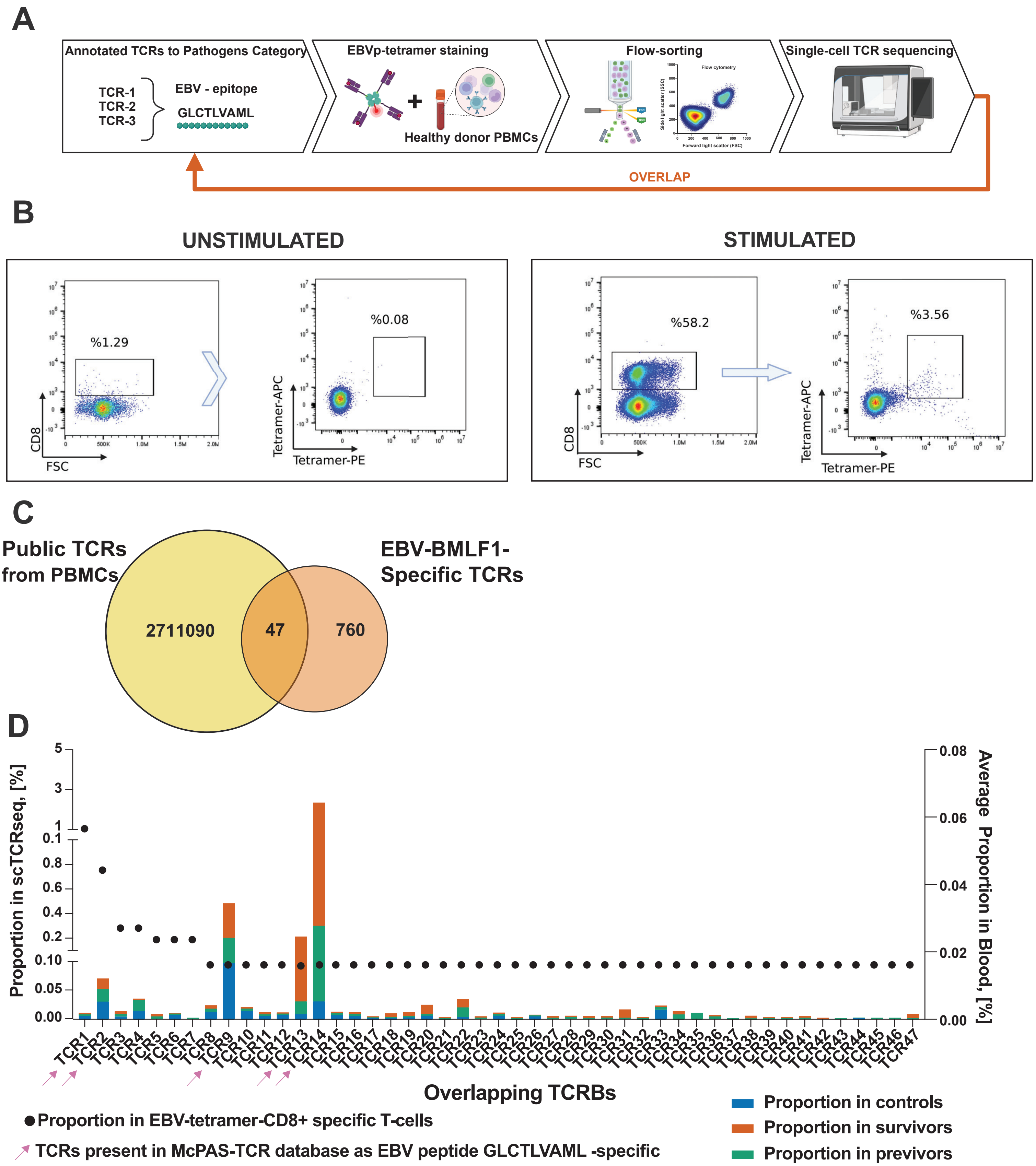

A
Annotated TCRs to Pathogens Category
TCR-1
TCR-2
TCR-3
EBV - epitope
GLCTLVAML
EBVp-tetramer staining
Healthy donor PBMCs
Flow-sorting
Single-cell TCR sequencing
OVERLAP
B
UNSTIMULATED
STIMULATED
%1.29
%0.08
%58.2
%3.56
CD8
FSC
Tetramer-APC
Tetramer-PE
C
Public TCRs from PBMCs
EBV-BMLF1-Specific TCRs
2711090
47
760
D
Proportion in scTCRseq, [%]
Average Proportion in Blood, [%]
Overlapping TCRBs
Proportion in EBV-tetramer-CD8+ specific T-cells
TCRs present in McPAS-TCR database as EBV peptide GLCTLVAML -specific
Proportion in controls
Proportion in survivors
Proportion in previvors

Figure S8. In vitro validation of public TCRβs annotated as EBV-specific. A, Schematic of the experimental set up; **B,** Flow cytometry images of the isolation EBV-tetramer-specific CD8$^+$ T-cells isolation before and after stimulation with the EBV peptide; **C,** Overlap of TCRs between the EBV-tetramer-specific CD8$^+$ T-cells and the public TCRβ cohort from blood/PBMCs; **D**, The X-axis shows each of the 47 TCRβs that overlapped between the EBV-tetramer-specific CD8$^+$ T-cells and our public TCRβ repertoire. The left Y-axis shows the Proportion within the EBV-tetramer-specific CD8$^+$ T-cells (black dots) and the right Y-axis shows the average proportion of those overlapping TCRs in the blood of our disease status groups (bars). The purple arrows highlight those TCRs that were previously identified as EBV-BMLF1-specific based on our annotation results against the McPAS-TCR database. Source data are provided as Source Data file Source Data.xlsx.

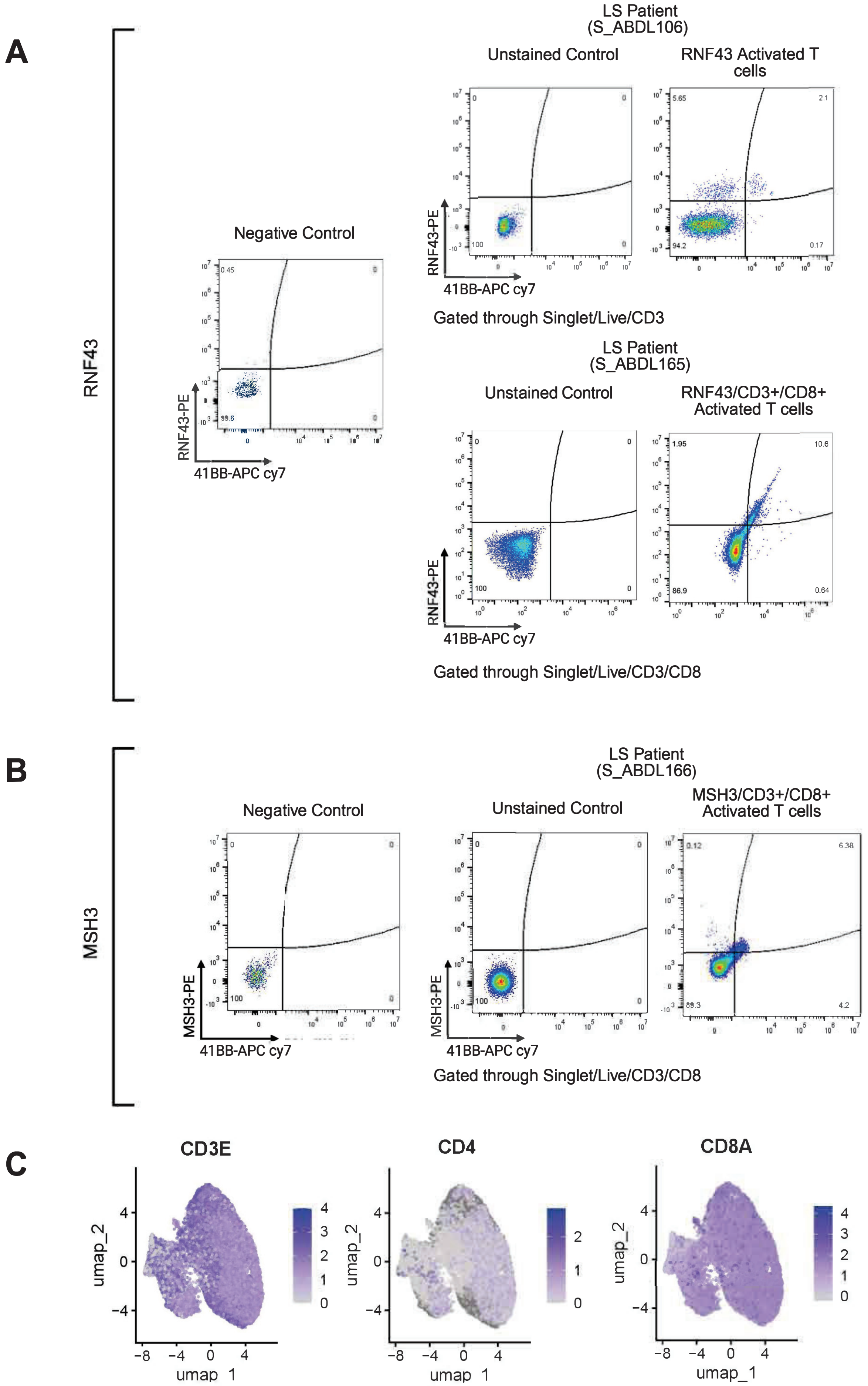
A
RNF43
Negative Control
RNF43-PE
41BB-APC cy7
LS Patient
(S_ABDL106)
Unstained Control
RNF43 Activated T cells
RNF43-PE
41BB-APC cy7
Gated through Singlet/Live/CD3
LS Patient
(S_ABDL165)
Unstained Control
RNF43/CD3+/CD8+ Activated T cells
RNF43-PE
41BB-APC cy7
Gated through Singlet/Live/CD3/CD8
B
MSH3
Negative Control
MSH3-PE
41BB-APC cy7
LS Patient
(S_ABDL166)
Unstained Control
MSH3/CD3+/CD8+ Activated T cells
MSH3-PE
41BB-APC cy7
Gated through Singlet/Live/CD3/CD8
C
CD3E
CD4
CD8A
umap_2
umap_1

Figure S9. Identification and UMAP projection of neoantigen-specific T cells from Lynch syndrome patients. A, Flow cytometry gating strategy for RNF43-dextramer-positive T cells from two LS patients. For LS carrier S_ABDL165 (top row), RNF43-specific activated $CD3^+$ T cells are shown following gating on singlets, live cells, and CD3. For LS carrier S_ABDL106 (bottom row), RNF43-specific activated $CD3^+CD8^+$ T cells are shown following additional CD8 gating. Controls in both cases include HLA-unmatched donor PBMCs (left) and unstained PBMCs for autofluorescence (middle); **B**, Gating strategy for MSH3-dextramer-positive T cells from LS carrier S_ABDL166. Controls include HLA-unmatched donor PBMCs (left) and unstained PBMCs (middle). MSH3-specific activated $CD3^+CD8^+$ T cells are shown on the right; **C**, UMAP projection of RNF43- and MSH3-dextramer-specific $CD3^+$ T cells from all three LS carriers (S_ABDL165, S_ABDL106, and S_ABDL166), annotated by $CD4^+$ and $CD8^+$ phenotypes.

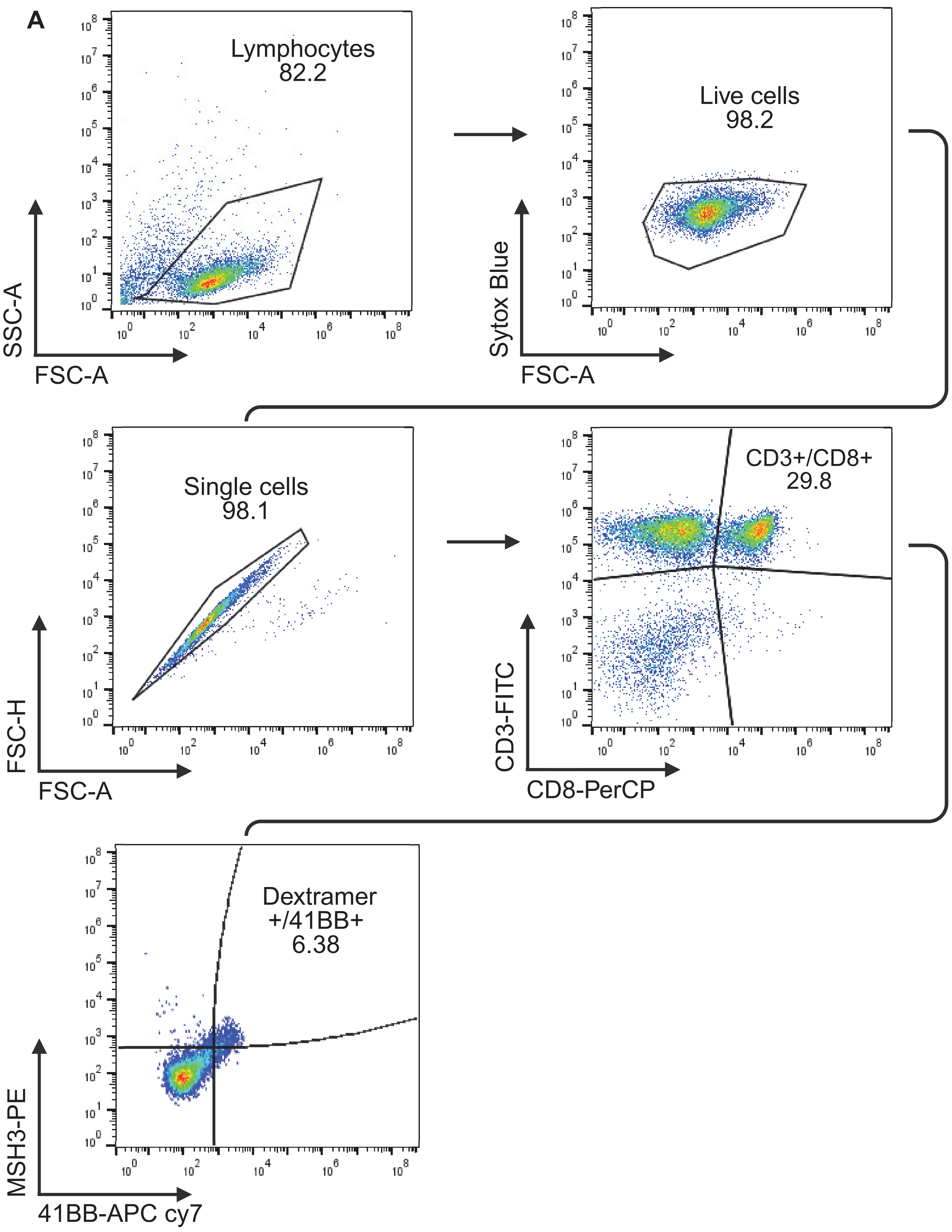

A
Lymphocytes
82.2
SSC-A
FSC-A
Live cells
98.2
Sytox Blue
FSC-A
Single cells
98.1
FSC-H
FSC-A
CD3+/CD8+
29.8
CD3-FITC
CD8-PerCP
Dextramer
+/41BB+
6.38
MSH3-PE
41BB-APC cy7

Figure S10. Flow cytometry gating strategy for identification of antigen-specific T cells. (A) Representative flow cytometry plots showing the sequential gating strategy used to identify antigen-specific T cells. Cells were sequentially gated on lymphocytes, singlets, $CD3^{+}/CD8^{+}$ T cells, and $dextramer^{+}/4\text{-}1BB^{+}$ cells, as indicated. Percentages shown represent the frequency of cells within each gated population.

Supplementary Table 1. Summary of colorectal tissue sample characteristics

Characteristic	n = 14	%
**Category**		
Precancer	8	58
Advanced Precancer	3	21
Cancer	3	21
**Pathology**		
Tubular Adenoma	11	79
Adenocarcinoma	3	21
**Type**		
FFPE	13	93
Frozen	1	7

Supplementary Table 2. Colorectal tissue sample cohort; MMRd, mismtach repair deficient, MMRp, mismatch repair proficient; NA, not assessed.

Sample ID	Tissue_type	Pathology	Matching PBMCs/Blood sample	Tissue Category	MMR Status	Year of Biopsy Collection	Year of Blood Collection
ADCA_ABT1	FFPE	Adenocarcinoma	S_ABDL167	Cancer	MMRd	2016	2020
TA_ABT2	FFPE	Tubular Adenoma	S_ABDL168	Precancer	MMRp	2017	2017
ADCA_ABT3	FFPE	Adenocarcinoma	S_ABDL166	Cancer	NA	2010	2020
TA_ABT4	Frozen	Tubular Adenoma	S_ABDL62	Precancer	NA	2020	2020
TA_ABT5	FFPE	Tubular Adenoma	S_ABDL184	Precancer	MMRd	2018	2019
TA_ABT6	FFPE	Tubular Adenoma	S_ABDL162	Advanced Precancer	Indeterminate	2013	2013
TA_ABT7	FFPE	Tubular Adenoma	S_ABDL166	Precancer	NA	2011	2020
ADCA_ABT8	FFPE	Adenocarcinoma	S_ABDL166	Cancer	MMRd	1996	2020
TA_ABT9	FFPE	Tubular Adenoma	S_ABDL166	Advanced Precancer	NA	2010	2020
TA_ABT10	FFPE	Tubular Adenoma	S_ABDL166	Precancer	MMRd	2012	2020
TA_ABT11	FFPE	Tubular Adenoma	P_ABDL201	Precancer	Indeterminate	2013	2013
TA_ABT12	FFPE	Tubular Adenoma	P_ABDL161	Advanced Precancer	NA	2013	2013
TA_ABT13	FFPE	Tubular Adenoma	P_ABDL171	Precancer	MMRp	2018	2018
TA_ABT14	FFPE	Tubular Adenoma	S_ABDL160	Precancer	MMRp	2016	2016

Supplementary Table 3. Gamma Generalized linear model output summary of the effect of different variables in the Simpson Clonality Index. The categories of each predictive variable that do not appear in the table were considered the reference levels. The intercept represents the baseline value of the Simpson index when all predictor variables in the model are held constant at their reference levels. Baseline reference for Disease Status = Survivors, Institution = MDA, Gender = Female, MMR gene = MLH1, Input Material = Blood, Age Range = >50 years old.

Simpson Index GLM Output Summary

Deviance Residuals

Min	1Q	Median	3Q	Max
-1.8641	-0.7885	-0.3311	0.2694	2.4192

**Predictors**	**Estimate**	**Std Error**	**t value**	***P***
(Intercept)	-2.9213	0.19075	-15.315	**< 2e-16**
Disease Status [Control]	-0.44603	0.20255	-2.202	**0.02852**
Disease Status [Previvor]	-0.34537	0.13259	-2.605	**0.00971**
Institution2 [KUCC]	-0.14059	0.19068	-0.737	0.46159
Institution2 [IDBELL]	-0.05986	0.20116	-0.298	0.76627
Gender [Male]	0.20151	0.11787	1.71	0.08852
MMRgene [MSH2]	-0.09415	0.15648	-0.602	0.54792
MMRgene [MSH6]	0.05731	0.17619	0.325	0.74523
MMRgene [PMS2]	0.18717	0.1961	0.954	0.34071
Input material [PBMCs]	0.08298	0.16609	0.5	0.61776
Age Range [≤50]	-0.36038	0.12098	-2.979	**0.00316**

Null deviance: 209.88 on 276 degrees of freedom

Residual deviance: 184.41 on 266 degrees of freedom

AIC: -1258.2

Number of Fisher Scoring iterations: 6

Supplementary Table 4. Gamma Generalized linear model output summary of the effect of different variables in the Simpson Clonality Index, focused on the HLA's effects from MDACC. The categories of each predictive variable that do not appear in the table were considered the reference levels. The intercept represents the baseline value of the Simpson index when all predictor variables in the model are held constant at their reference levels. Baseline reference for Disease Status = Control, Gender = Female, MMR gene = MLH1, Input Material = Blood, Age Range = >50 years old; Note: Top 10 frequent HLA were selected with ties (11 HLAs were selected).

	**Simpson_index**		
**Predictors**	**Estimates**	**CI**	***P***
**(Intercept)**	**0.01**	**0.01 – 0.04**	**<0.001**
Status MMRd Cancer [Previvor]	1.52	0.86 – 2.72	0.128
**Status MMRd Cancer [Survivor]**	**1.98**	**1.14 – 3.50**	**0.012**
Gender [Male]	1.04	0.75 – 1.45	0.798
MMRgene [MSH2]	0.68	0.42 – 1.09	0.106
MMRgene [MSH6]	0.84	0.50 – 1.42	0.508
MMRgene [PMS2]	1.21	0.66 – 2.25	0.518
Input material [PBMCs]	1.1	0.70 – 1.71	0.662
**Age Range3 [≤50]**	**0.65**	**0.46 – 0.93**	**0.014**
A*01 × 01TRUE	0.72	0.48 – 1.08	0.095
A*02 × 01TRUE	0.95	0.66 – 1.36	0.77
C*07 × 01TRUE	1.24	0.76 – 2.08	0.36
DQA1*05 × 01TRUE	1.01	0.62 – 1.69	0.949
DQB1*03 × 01TRUE	1.43	0.98 – 2.09	0.054
DPA1*01 × 03TRUE	1.98	0.81 – 4.48	0.097
A*03 × 01TRUE	1.06	0.70 – 1.62	0.791
C*07 × 02TRUE	1.04	0.68 – 1.63	0.836
DQA1*01 × 02TRUE	1.19	0.80 – 1.79	0.355
DPA1*02 × 01TRUE	1.18	0.81 – 1.75	0.392
DPB1*04 × 01TRUE	1.1	0.74 – 1.61	0.604
Observations	157		
R^2 Nagelkerke	0.236		
