# Supplementary material for: Genomic analysis of T Cell receptors reveals lynch syndrome specific immune signatures
Source: Nat Commun. 2026 Apr 3;17:4790. doi: 10.1038/s41467-026-71243-z (PMC13219734; doi:10.1038/s41467-026-71243-z)
Supplement: Supplementary file 2 — Description of Additional Supplementary Files [file 41467_2026_71243_MOESM2_ESM.pdf]

Description of Additional Supplementary Files

Supplementary Data 1: AUROC values of the LS classifier's performance based on overlapping TCRβ segments.

Supplementary Data 2: Selection of validation and training/testing sets.

Supplementary Data 3: Metadata obtained from the public dataset syn61987835.

Supplementary Data 4: Lynch Syndrome carrier-associated TCR signature (combining LS previvors and survivors).

Supplementary Data 5: AUROC values of previvor classifier's performance based on different classifiers and parameters.

Supplementary Data 6: LS previvor-associated TCR signature.

Supplementary Data 7: AUROC values of survivor classifier's performance based on overlapping TCRβ segments.

Supplementary Data 8: LS survivor-associated TCR signature.

Supplementary Data 9: TCRβ sequences of 47 TCRβs that overlapped between EBV-tetramerspecific $CD8^+$ T cells and our circulating public TCRβ cohort.

Supplementary Data 10: TCRs that overlapped between the Lynch Syndrome-associated TCRβ signature and RNF43-$tetramer^+$ $CD3^+$ T cells.

Supplementary Data 11: TCRs that overlapped between the Lynch Syndrome-associated TCRβ signature and the MMRd-CRC-resident TCR pool. Highly expanded TCRs are noted in bold.

Supplementary Data 12: PBMCs and whole blood sample cohort.
