# Supplementary material for: Genomic analysis of T Cell receptors reveals lynch syndrome specific immune signatures
Source: Nat Commun. 2026 Apr 3;17:4790. doi: 10.1038/s41467-026-71243-z (PMC13219734; doi:10.1038/s41467-026-71243-z)
Supplement: Supplementary file 15 — Reporting Summary [file 41467_2026_71243_MOESM15_ESM.pdf]

nature portfolio

Corresponding author(s): Eduardo Vilar
Last updated by author(s): Jan 21, 2026

# Reporting Summary

Nature Portfolio wishes to improve the reproducibility of the work that we publish. This form provides structure for consistency and transparency in reporting. For further information on Nature Portfolio policies, see our Editorial Policies and the Editorial Policy Checklist.

## Statistics

For all statistical analyses, confirm that the following items are present in the figure legend, table legend, main text, or Methods section.

| n/a | Confirmed | |
|---|---|---|
| ☐ | ☒ | The exact sample size (*n*) for each experimental group/condition, given as a discrete number and unit of measurement |
| ☐ | ☒ | A statement on whether measurements were taken from distinct samples or whether the same sample was measured repeatedly |
| ☐ | ☒ | The statistical test(s) used AND whether they are one- or two-sided<br>*Only common tests should be described solely by name; describe more complex techniques in the Methods section.* |
| ☐ | ☒ | A description of all covariates tested |
| ☐ | ☒ | A description of any assumptions or corrections, such as tests of normality and adjustment for multiple comparisons |
| ☐ | ☒ | A full description of the statistical parameters including central tendency (e.g. means) or other basic estimates (e.g. regression coefficient) AND variation (e.g. standard deviation) or associated estimates of uncertainty (e.g. confidence intervals) |
| ☐ | ☒ | For null hypothesis testing, the test statistic (e.g. *F*, *t*, *r*) with confidence intervals, effect sizes, degrees of freedom and *P* value noted<br>*Give P values as exact values whenever suitable.* |
| ☒ | ☐ | For Bayesian analysis, information on the choice of priors and Markov chain Monte Carlo settings |
| ☒ | ☐ | For hierarchical and complex designs, identification of the appropriate level for tests and full reporting of outcomes |
| ☐ | ☒ | Estimates of effect sizes (e.g. Cohen's *d*, Pearson's *r*), indicating how they were calculated |

*Our web collection on statistics for biologists contains articles on many of the points above.*

## Software and code

Policy information about availability of computer code

| | |
|---|---|
| Data collection | TCRseq data were collected by Adaptive Biotechnologies. |
| Data analysis | ComplexHeatmap v2.8.0, and Seurat v5.1.0. Code is available at https://zenodo.org/records/131410527<br>preview=l&token=eyJhbGciOiJIUzUxMiJ9.eyJpZCl61mY0M2VjNjEzLTZiNzAtNGVhOSliMmM1LWEOMjBiNGYzZDdkOClslmRhdGEiOnt9LCJyYW5k<br>b20iOiJkOWNIZDlhZTA2OWlzNDQxMWRjODdkMDBjMDQ10TUwNyJ9.-xwtuzKu9Nbx31-<br>rkUleNNdU14aGZTr0WNrnlssNHsARZgdanwTPXGr9T7L_8ufrlVbgwXVenXiTWGEQrHmuQ.<br>Computer code can be available at : https://github.com/Vilarlab-MDACC/LS_TCR |

For manuscripts utilizing custom algorithms or software that are central to the research but not yet described in published literature, software must be made available to editors and reviewers. We strongly encourage code deposition in a community repository (e.g. GitHub). See the Nature Portfolio guidelines for submitting code & software for further information.

# Data

Policy information about availability of data

All manuscripts must include a data availability statement. This statement should provide the following information, where applicable:

- Accession codes, unique identifiers, or web links for publicly available datasets
- A description of any restrictions on data availability
- For clinical datasets or third party data, please ensure that the statement adheres to our policy

The TCRseq data generated in this study are publicly available in Zenodo. To review it go to:
https://zenodo.org/records/13141052?
preview=1&token=eyJhbGciOiJIUzUxMiJ9.eyJpZCI6ImY0M2VjNjEzLTZiNzAtNGVhOS1iMmM1LWE0MjBiNGYzZDdkOCIsImRhdGEiOnt9LCJyYW5kb20iOiJkOWNlZDlhZTA2OWIzNDQxMWRjODdkMDBjMDQ1OTUwNyJ9.-xwtuzKu9Nbx3I-rkUIeNNdU14aGZTr0WfVrnLssNHsARZgdanwTPXGr9T7L_8ufrlVbgwXVenXiTWGEQrHmuQ

# Research involving human participants, their data, or biological material

Policy information about studies with human participants or human data. See also policy information about sex, gender (identity/presentation), and sexual orientation and race, ethnicity and racism.

| | |
|---|---|
| Reporting on sex and gender | In this study, sex was considered as a potential covariate, but we did not perform separate analyses by sex. Sex was included as a predictor variable in our generalized linear model to account for any potential sex-based differences in the Simpson clonality index of the TCR repertoires. By including this covariate in the model, we controlled for its possible effects on the outcome variable. This approach allowed us to examine the overall influence of sex while maintaining the primary focus on the TCR signature based on disease status. |
| Reporting on race, ethnicity, or other socially relevant groupings | Race was provided as a descriptive characteristic of the population cohort in Table 1, but was not included in any analysis. |
| Population characteristics | The human research participants in this study consisted of three groups: Lynch syndrome survivors (n=102), Lynch syndrome previvors (n=130), and controls (n=45), all of whom were carefully characterized with respect to key covariates in Table 1. The groups were assessed for sex, age, institution of recruitment, and genetic information, particularly focusing on the MMR gene carrying a germline pathogenic variant. Additionally, we considered the input material (blood vs. PBMCs) as a covariate. |
| Recruitment | All LS individuals included in this study had a confirmed diagnosis of Lynch Syndrome by germline genetic testing and were recruited during their regular standard of care surveillance visits and procedures at MD Anderson Cancer Center, The University of Kansas Cancer Center, and the Institut Català d'Oncologia (Barcelona, Spain). Controls were recruited during their average-risk routine colonoscopy screening starting at age 45. |
| Ethics oversight | MD Anderson Cancer Center Institutional Review Board, IRB #PA12-0327 , IRB #PA13-0178 |

Note that full information on the approval of the study protocol must also be provided in the manuscript.

# Field-specific reporting

Please select the one below that is the best fit for your research. If you are not sure, read the appropriate sections before making your selection.

☒ Life sciences ☐ Behavioural & social sciences ☐ Ecological, evolutionary & environmental sciences

For a reference copy of the document with all sections, see nature.com/documents/nr-reporting-summary-flat.pdf

# Life sciences study design

All studies must disclose on these points even when the disclosure is negative.

| | |
|---|---|
| Sample size | Our sample size of 102 LS survivors, 130 LS previvors, and 45 controls was chosen based on sample availability and cost of sequencing, aiming to include sufficient participants to detect meaningful differences, particularly between LS individuals and controls. We were able to achieve statistically significant results differentiating LS individuals from controls, suggesting that the sample size was adequate for this analysis. However, for differentiating LS previvors from survivors, the sample size was not large enough to detect significant differences. This highlights the need for a larger sample size to detect more subtle differences between these subgroups, which we acknowledge as a limitation of the current study. |
| Data exclusions | Only samples that were collected but did not yield enough DNA for TCR sequencing, were excluded and not accounted in the study. |
| Replication | We did not perform external replication due to the nature of the analysis, which required a large and diverse dataset to develop robust models. Instead, we focused on leveraging the existing data. However, we randomly selected 20% of the samples as a validation set to evaluate the markers we identified. This approach provided a thorough assessment of model stability and allowed us to validate the results |

within the study cohort.

Randomization

In this study, sample allocation was not random, as participants were grouped based on their clinical status (LS survivors, LS previvors, and controls). Since this was a proof-of-concept study and needed each group to contain as many samples as possible, these were the only categories assessed. To account for potential confounding effects of covariates, we determined the effects of age, institution, gender, the MMR gene with the germline pathogenic variant, and the input material (blood vs. PBMCs) on the diversity of the samples. For this, we applied a generalized linear model for gamma-distributed data and evaluated the correlation between age and the Simpson clonality index using Spearman's rank correlation coefficient.

Blinding

In this study, blinding was not considered necessary because the analysis focused on identifying TCR signatures across different participant groups (LS survivors, LS previvors, and controls). The outcome measures were objective and not subject to interpretation. Moreover, group classifications were pre-defined, and the study design did not involve decisions that could be influenced by knowledge of group membership. Therefore, blinding was not required to minimize bias in the analysis.

# Reporting for specific materials, systems and methods

We require information from authors about some types of materials, experimental systems and methods used in many studies. Here, indicate whether each material, system or method listed is relevant to your study. If you are not sure if a list item applies to your research, read the appropriate section before selecting a response.

## Materials & experimental systems

| n/a | Involved in the study | |
|---|---|---|
| ☐ | ☒ | Antibodies |
| ☒ | ☐ | Eukaryotic cell lines |
| ☒ | ☐ | Palaeontology and archaeology |
| ☒ | ☐ | Animals and other organisms |
| ☒ | ☐ | Clinical data |
| ☒ | ☐ | Dual use research of concern |
| ☒ | ☐ | Plants |

## Methods

| n/a | Involved in the study | |
|---|---|---|
| ☒ | ☐ | ChIP-seq |
| ☐ | ☒ | Flow cytometry |
| ☒ | ☐ | MRI-based neuroimaging |

## Antibodies

Antibodies used

R-phycoerythrin (PE)–labeled pMHC tetramer (ProImmune), specific for BMLF1-EBV (GLCTLVAML), HLA-A02:01, and allophycocyanin (APC)–labeled pMHC tetramer (ProImmune), specific for BMLF1-EBV (GLCTLVAML), HLA-A02:01, were used at a fixed volume per sample according to the manufacturer's staining protocol. PE-labeled pMHC dextramer (Immudex), specific for RNF43 (TQLARFFPI), HLA-A*02:01, was used at a fixed volume per sample according to the manufacturer's staining protocol. For surface staining, cells were incubated with Peridinin-Chlorophyll-Protein Complex (PerCP)–labeled mouse anti-human CD8 (BD Biosciences, Cat# 347314) at a 1:50 dilution, Fluorescein Isothiocyanate (FITC)–labeled mouse anti-human CD3 (BD Biosciences, Cat# 561807) at a 1:50 dilution, and Allophycocyanin-Cyanine7 (APC-Cy7)–labeled mouse anti-human 4-1BB (BioLegend, Cat# 309830) at a 1:50 dilution. All antibodies were used according to the manufacturers' recommendations.

Validation

Validation of pMHC tetramer and dextramer staining, as well as antibody staining, was performed in accordance with manufacturer-recommended guidelines and established best practices (Immudex: https://www.immudex.com/dextramer-staining-protocol; ProImmune: https://www.proimmune.com/protocols). Multiple controls were used to ensure specificity and minimize background signal, including HLA-mismatched donor PBMCs as negative controls to confirm antigen-specific staining and exclude non-specific binding. Unstained controls were included to assess background fluorescence and to establish appropriate gating thresholds

## Plants

Seed stocks

N/A

Novel plant genotypes

N/A

Authentication

N/A

## Flow Cytometry

### Plots

Confirm that:

☒ The axis labels state the marker and fluorochrome used (e.g. CD4-FITC).

☒ The axis scales are clearly visible. Include numbers along axes only for bottom left plot of group (a 'group' is an analysis of identical markers).

☒ All plots are contour plots with outliers or pseudocolor plots.

☒ A numerical value for number of cells or percentage (with statistics) is provided.

### Methodology

| | |
|---|---|
| Sample preparation | Peripheral blood mononuclear cells (PBMCs) were isolated from blood samples of healthy donors and Lynch Syndrome (LS) patients using density gradient centrifugation (Ficoll-Paque). Cells were cultured in R10 medium (RPMI 1640 with 10% FBS, 10 mM Hepes, and 1X Pen/Strep) supplemented with cytokines (IL-2, IL-7, IL-15, GM-CSF, and Flt-3L, depending on the experiment). Peptide stimulation was performed using either BMLF1 (GLCTLVAML, EBV-specific) or RNF43_3 (TQLARFFPI, neoantigen-specific) peptides. Cells were expanded over 12 days, with intermittent cytokine feeding. After stimulation, antigen-specific T cells were sorted using tetramers (for viral specificity) or dextramers (for neoantigen specificity) before undergoing single-cell RNA and TCR sequencing. |
| Instrument | Flow Cytometry: CytoFLEX SRT (Beckman Coulter, USA) |
| Software | Flow Cytometry Analysis: FlowJo v10.8.1 |
| Cell population abundance | The post-sort purity of isolated T lymphocytes and antigen-specific T cells was assessed using flow cytometry. Lymphocyte purity in PBMC samples varied between 68% and 85%, while the unstimulated CD3+ T cell population was confirmed to be >96% pure using CD3-FITC staining. Antigen-specific cells were considered positive if they exhibited >1% positivity following tetramer or dextramer staining. After antigen-specific enrichment:EBV-BMLF1 tetramer-positive CD8+ T cells comprised 3.56% of total CD8+ T cells. RNF43-dextramer-positive CD3+ T cells comprised 2.1% of total CD3+ T cells. |
| Gating strategy | Lymphocyte populations were first identified based on their small size and low granularity using FSC-A vs. SSC-A gating, allowing for the exclusion of debris and non-lymphoid cells. To ensure single-cell analysis, doublets and aggregates were removed by applying FSC-H vs. FSC-A and SSC-H vs. SSC-A gating, selecting cells that displayed a linear relationship characteristic of single cells.Live/dead discrimination was performed using Sytox Blue staining to exclude dead cells. T-cell populations were then identified, with CD3+ T cells selected using CD3-FITC staining and further confirmed through SSC-A vs. CD3-FITC plots.For antigen-specific selection:Viral-specific T cells were identified by gating CD8+ T cells that were dual-positive for PE and APC EBV-BMLF1 tetramers.Neoantigen-specific T cells were identified by gating CD3+ T cells that were RNF43-dextramer-positive, with additional refinement using CD8 and 41BB markers.As a final purity assessment step, unstained PBMCs were used to evaluate autofluorescence and background fluorescence. HLA-mismatched PBMCs served as negative controls to establish gating boundaries. Fluorescence intensity distributions were compared against negative controls to ensure precise and accurate population separation. |

☒ Tick this box to confirm that a figure exemplifying the gating strategy is provided in the Supplementary Information.
